# Fission Yeast Exo1 and Rqh1-Dna2 Redundantly Contribute to Resection of Uncapped Telomeres

**DOI:** 10.1371/journal.pone.0140456

**Published:** 2015-10-14

**Authors:** Tomoko Nanbu, Luân C. Nguyễn, Ahmed G. K. Habib, Naoya Hirata, Shinobu Ukimori, Daiki Tanaka, Kenta Masuda, Katsunori Takahashi, Masashi Yukawa, Eiko Tsuchiya, Masaru Ueno

**Affiliations:** 1 Department of Molecular Biotechnology, Graduate School of Advanced Sciences of Matter, Hiroshima University, 1-3-1 Kagamiyama, Higashi-Hiroshima, 739-8530, Japan; 2 Research Center for the Mathematics on Chromatin Live Dynamics, Hiroshima University, 1-3-1 Kagamiyama, Higashi-Hiroshima, 739-8530, Japan; Tulane University Health Sciences Center, UNITED STATES

## Abstract

The uncapping of telomeres induces a DNA damage response. In *Schizosaccharomyces pombe*, deletion of *pot1* ^*+*^ causes telomere uncapping and rapid telomere resection, resulting in chromosome fusion. Using the *nmt-pot1-aid* strain, we previously reported that Pot1 shut-off causes telomere loss and chromosome fusion in *S*. *pombe*. However, the factors responsible for the resection of uncapped telomeres remain unknown. In this study, we investigated these factors and found that concomitant deletion of *rqh1* ^*+*^ and *exo1* ^*+*^ alleviated the loss of telomeres following Pot1 shut-off, suggesting that Rqh1 and Exo1 are redundantly involved in the resection of uncapped telomeres. We also investigated the role of Rqh1 helicase activity and found it to be essential for the resection of uncapped telomeres. Moreover, we found that Dna2 and Exo1 function redundantly in the resection of uncapped telomeres. Taken together, these results suggest that Exo1 and Rqh1-Dna2 redundantly contribute to the resection of uncapped telomeres. Therefore, our results demonstrate that *nmt-pot1-aid* is an important model strain to study the role of helicases and nucleases in the resection of uncapped telomeres and to improve our understanding of DNA double-strand break repair.

## Introduction

Telomeres are protected by telomere-capping proteins [1]. As a consequence of critically short telomere or dysfunction of telomere-capping proteins, telomeres become uncapped and are recognized as double-strand breaks (DSB) [2–4]. In *Saccharomyces cerevisiae*, a 5´ to 3´ exonuclease, Exo1, and a RecQ helicase, Sgs1, are redundantly involved in DSB resection [5]. Similarly, Exo1 and the RecQ helicase, Rqh1, mediate DSB resection pathways in *Schizosaccharomyces pombe* [6, 7]. In *S*. *cerevisiae*, inactivation of the telomere-capping protein Cdc13 by the temperature sensitive *cdc13-1* allele results in telomere uncapping [8]. Although resection of *cdc13-1*-induced uncapped telomeres also involves both Exo1 and Sgs1 activity, Exo1 plays more important role than Sgs1 [9–11]. In *S*. *pombe*, the protection of telomere protein 1 (Pot1) plays an essential role in telomere capping and is thought to be a functional homologue of Cdc13 in *S*. *cerevisiae* [12]. Similarly, inactivation of Pot1 by using temperature sensitive *pot1* allele results in telomere uncapping [13]. Recently, Exo1 was identified as a suppressor of the *pot1* ts allele, suggesting that Exo1 is involved in the resection of uncapped telomeres in *S*. *pombe* [14]. However, it is still not clear whether Rqh1 is also involved in the resection of uncapped telomeres in *S*. *pombe*.

In a previous study, we showed that the *S*. *pombe nmt-pot1-aid* strain, which contains the thiamine-repressive *nmt81* promoter and auxin-inducible degron (*aid*) tag, loses the majority of its telomere signal after Pot1 is shut off in the presence of thiamine and auxin [15]. However, the mechanisms involved in this process are yet to be elucidated. Therefore, in this study, we investigated the potential factors responsible for this resection, specifically the involvement of nucleases and helicases. Our results suggest that resection of uncapped telomeres involves Rqh1-Dna2 and Exo1, acting redundantly.

## Results

### Deletion of both *rqh1* ^*+*^ and *exo1* ^*+*^ alleviates the loss of telomeres following Pot1 shut-off

**Fig 1:**
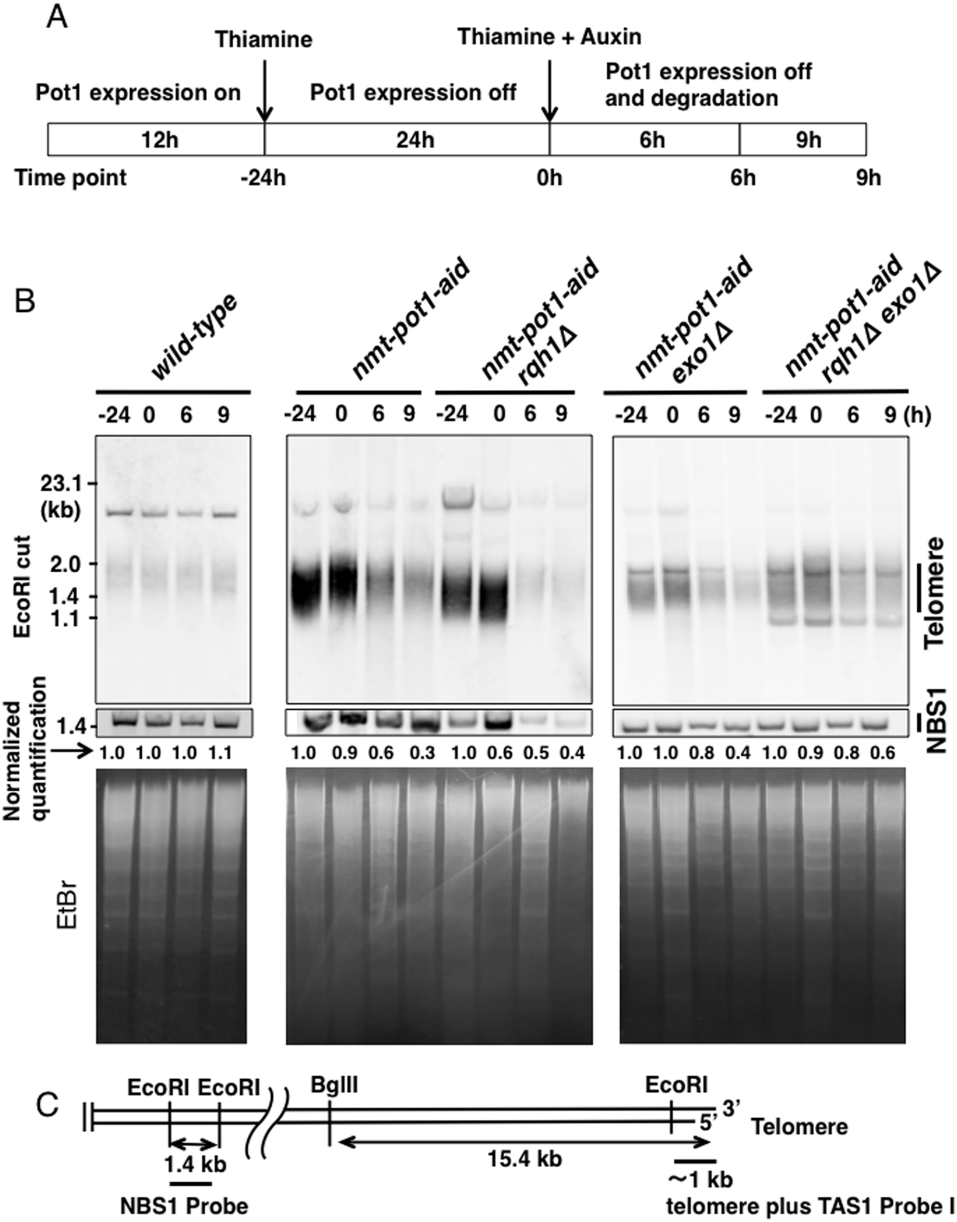
Double deletion of *rqh1* ^*+*^ and *exo1* ^*+*^ alleviates the telomere loss following Pot1 shut-off. **(A)** Experimental design to show how Pot1 function is shut-off. Cells were cultured in EMM medium for 12h without thiamine (time point −24 h). Next, cells were pre-incubated with 15 μM thiamine for 24 h to reduce expression of Pot1 (time point 0 h). Subsequently, both 15 μM thiamine and auxin (0.5 mM of 1-naphtaleneacetic acid) were added and incubated for another 6 h and 9 h (time points: 6 h and 9 h). **(B)** Telomere length was analyzed using Southern hybridization. Wild-type, *nmt-pot1-aid*, *nmt-pot1-aid rqh1Δ*, *nmt-pot1-aid exo1Δ*, and *nmt-pot1-aid rqh1Δ exo1Δ* strains were used. Genomic DNA was digested with EcoRI, and resolved by 1.5% agarose gel electrophoresis. A 1-kb DNA fragment containing telomeric DNA plus telomere-associated sequence 1 (TAS1) was used for hybridization (see C). The normalized quantification value of the telomere band is shown below the Southern hybridization data. Image J was used for quantitation. The intensity of the telomere bands was divided by the intensity of the 1.4-kb NBS1 band, located 1.9 Mb from the right telomere in chromosome II, to adjust for loaded DNA fragments (see C). Additionally, in each strain, the band intensity at time point −24 h was normalized to 1. To assess the total amount of DNA, the gel was stained with EtBr, before blotting on to the membrane. (**C**) Restriction enzyme sites of chromosome ends cloned in the plasmid pNSU70 [42]. The location of the probe used for hybridization is shown by a thick bar. Primers used for amplification of the NBS1 probe are shown in Table 2.

Previously, we showed that deletion of *rqh1* ^*+*^ does not affect telomere loss in the *nmt-pot1-aid* strain of *S*. *pombe* following Pot1 shut-off in the presence of thiamine and auxin [15]. This finding suggests that either Rqh1 is not involved in telomere resection or Rqh1 is involved in resection but another redundant pathway–possibly mediated by Exo1–can resect uncapped telomeres, in the absence of Rqh1. We investigated these hypotheses by deleting both *rqh1* ^*+*^ and *exo1* ^*+*^ in the *nmt-pot1-aid* strain of *S*. *pombe*. We first pre-incubated both *nmt-pot1-aid exo1Δ* cells and *nmt-pot1-aid rqh1Δ exo1Δ* cells in the presence of thiamine for 24 h to reduce the expression of *pot1* ^*+*^ (time point 0 h). Both thiamine and auxin were then added and cells were incubated for 6 h and 9 h, as previously described (Fig 1A) [15]. The telomere band, digested with EcoRI, was quantified and normalized by the intensity of the 1.4-kb NBS1 band to adjust for loaded DNA fragments (Fig 1B and 1C). Deletion of *exo1* ^*+*^ in the *nmt-pot1-aid* strain slightly affected the telomere loss following Pot1 shut-off (Fig 1B and 1C). Interestingly, concomitant deletion of *rqh1* ^*+*^ and *exo1* ^*+*^ alleviated telomere loss at time points: 6 h and 9 h, suggesting that Rqh1 and Exo1 function redundantly in the resection of uncapped telomeres.

**Fig 2:**
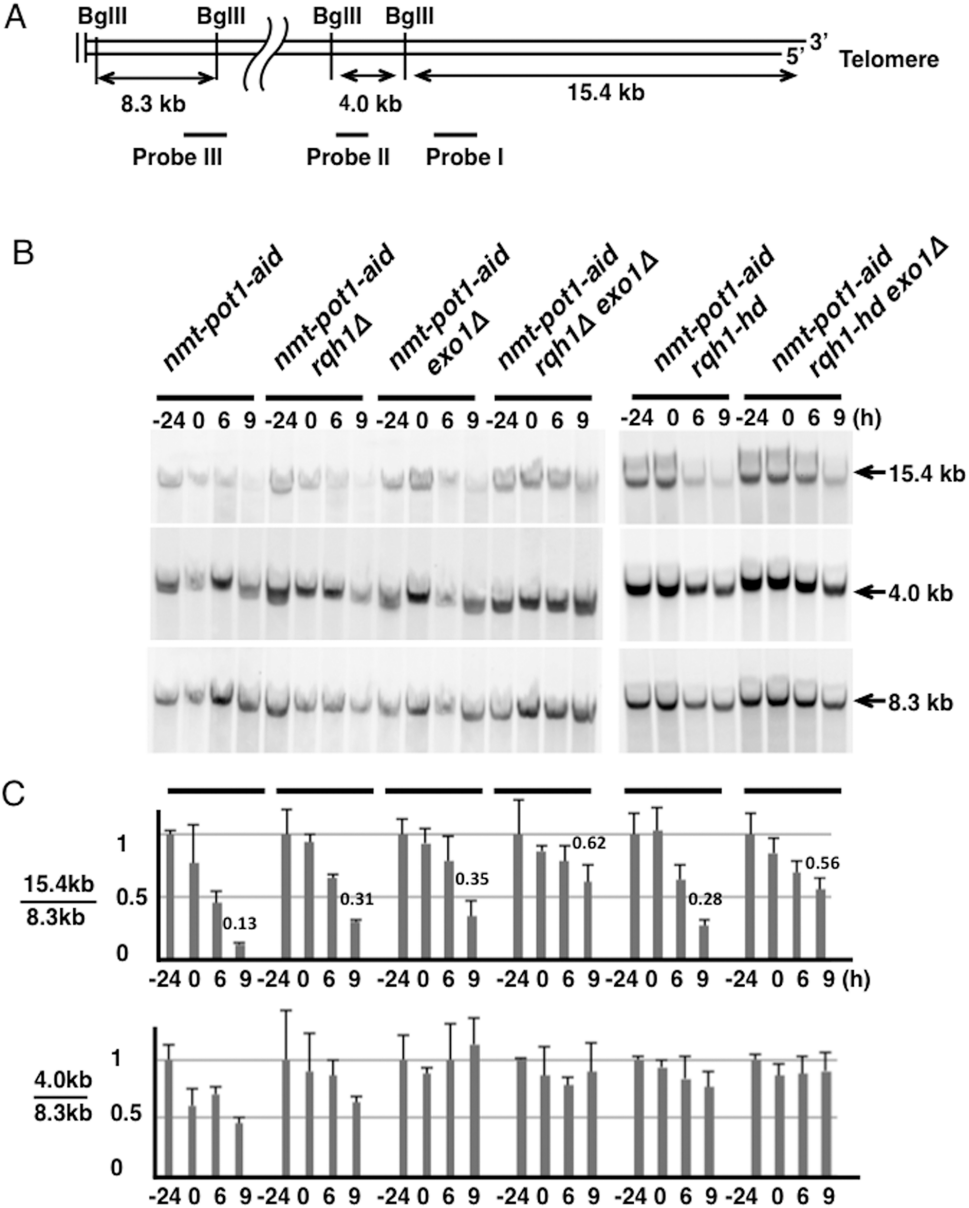
Quantitation of telomere resection following Pot1 shut-off shows that Rqh1 and Exo1 resect uncapped telomere redundantly. (**A**) BglII site of the terminal region on chromosomes I and II [28]. BglII sites located about 1.9 Mbp from the telomere end, on the right arm of chromosome II, are also shown. The positions of the three probes (I, II, and III) that were used to detect BglII-digested fragments are indicated. Primers used for amplification of these probes are shown in Table 2. (**B**) The telomere resection was analyzed using Southern hybridization. The *nmt-pot1-aid*, *nmt-pot1-aid rqh1Δ*, *nmt-pot1-aid exo1Δ*, *nmt-pot1-aid rqh1Δ exo1Δ*, *nmt-pot1-aid rqh1-hd*, and *nmt-pot1-aid rqh1-hd exo1Δ* strains were used. Cells were incubated as described in Fig 1. Genomic DNA was digested with BglII and separated by 0.5% agarose gel electrophoresis. The 3 probes (I, II, and III) shown in (A) were used to detect BglII-digested fragments. (**C**) Quantitation of the band intensity shown in (B). The band was quantitated as shown in Fig 1B. The intensity of the 15.4- and 4.0-kb bands was divided by the intensity of the 8.3-kb band to adjust for loaded DNA fragments. Data represent the mean and standard deviation of 2 to 5 independent experiments. For comparison, the normalized quantitation value of the telomere loss at time point 9 h is shown above the bar graph.

Next, we investigated telomere resection using genomic DNA digested with BglII, which produced a 15.4-kb telomere fragment and an adjacent 4.0-kb fragment (Fig 2A). An 8.3-kb fragment, located 1.9 Mb from the right telomere in chromosome II, was visualized as the internal control. As expected, we detected the 15.4-kb telomere fragment in the *nmt-pot1-aid* strain (Fig 2B). However, a significant signal loss was observed in this fragment after 9 h incubation following Pot1 shut-off (Fig 2B and 2C). Although the single deletion of either *rqh1* ^*+*^ or *exo1* ^*+*^ did not significantly affect the loss of the 15.4-kb fragment, concomitant deletion of *rqh1* ^*+*^ and *exo1* ^*+*^ alleviated signal loss (Fig 2B and 2C). This result provides further evidence that Rqh1 and Exo1 function redundantly in the resection of uncapped telomeres.

The helicase activity of Sgs1 is known to be essential for the resection of DSB ends [16–18]. Therefore, we investigated whether the helicase activity of Rqh1 is similarly essential for the resection of uncapped telomeres. We used the helicase-dead point mutant *rqh1-hd*, which shows no helicase activity in vitro [19]. Although the single mutation of *rqh1-hd* did not affect resection in the *nmt-pot1-aid* strain following Pot1 shut-off, the *rqh1-hd exo1Δ* double mutation alleviated the signal loss in the 15.4-kb fragment (Fig 2B and 2C). These results indicate that the helicase activity of Rqh1 is essential for the resection of uncapped telomeres.

We also quantitated the band intensity of the 4.0-kb adjacent fragment, which was also reduced in the *nmt-pot1-aid* strain after Pot1 shut-off. We found that deletion of *rqh1* ^*+*^ did not significantly affect the signal loss in the 4.0-kb band, but deletion of *exo1* ^*+*^ suppressed the signal loss (Fig 2B and 2C). This suggests that Exo1 plays a more important role than Rqh1 in long-range resection of uncapped telomeres.

### Double deletion of *rqh1* ^*+*^ and *exo1* ^*+*^ suppresses the loss of NotI-digested chromosome-end fragments and viability following Pot1 shut-off

**Fig 3:**
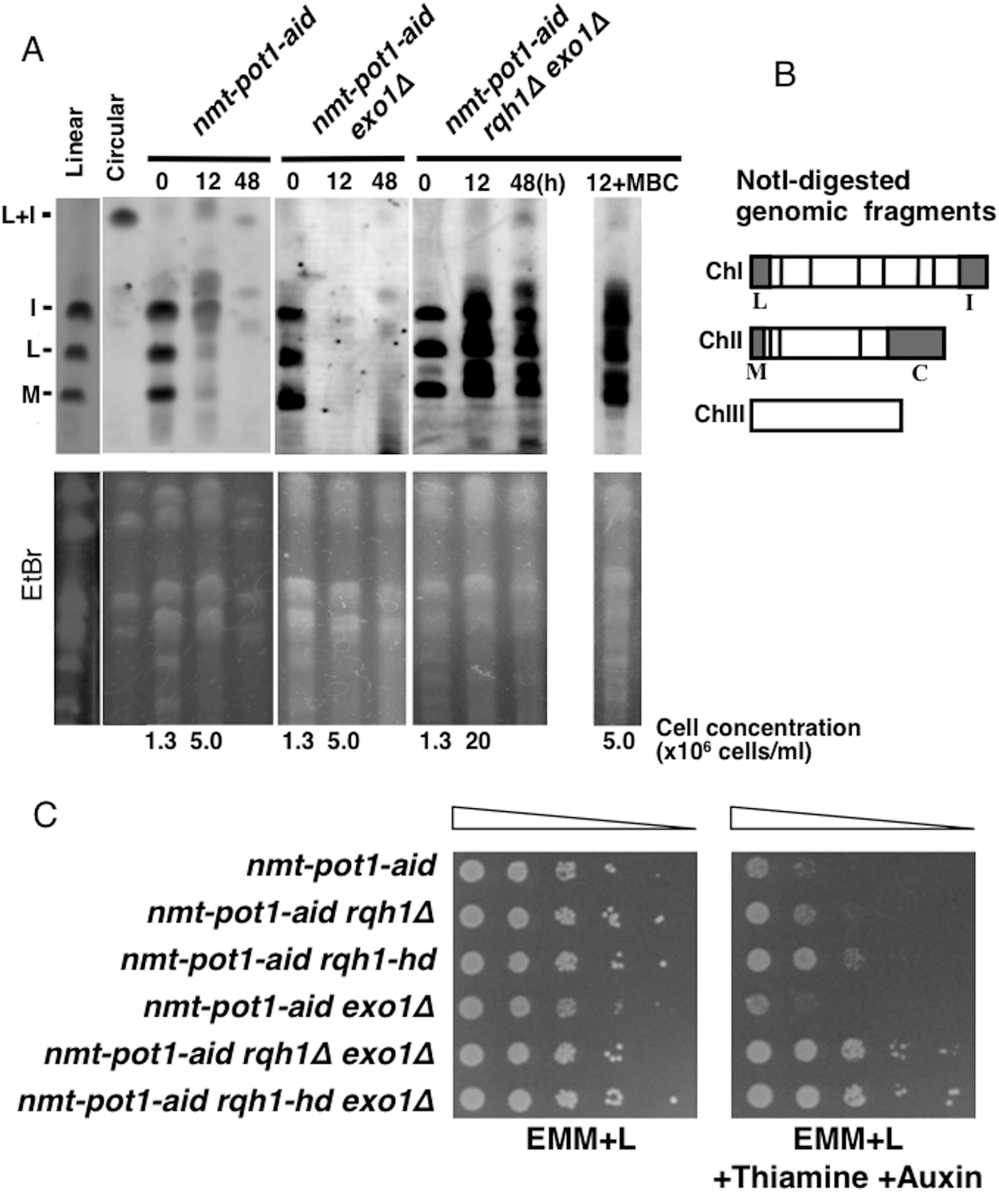
Double deletion of *rqh1* ^*+*^ and *exo1* ^*+*^ suppresses the loss of NotI-digested chromosome-end fragments and viability following Pot1 shut-off. (**A**) The NotI-digested *S*. *pombe* chromosomal DNA was analyzed by PFGE. The *nmt-pot1-aid*, *nmt-pot1-aid exo1Δ*, and *nmt-pot1-aid rqh1Δ exo1Δ* strains were used. Strains with linear (wild-type JY741) and circular chromosomes (*pot1Δ* KTA045) were used as controls [15]. Cells were incubated as described in Fig 1, except that cells were incubated with thiamine and auxin for 12 h and 48 h. For the *nmt-pot1-aid rqh1Δ exo1Δ* strain, 5μg/ml of carbendazim (MBC) was also added at time point 0 h to arrest the cell cycle when indicated (12 h +MBC). Cell concentration at time point 0 h and 12 h is shown below the EtBr data. We used probes specific for the NotI fragments (I, L, and M) [20]. To assess the total amount of DNA, the gel was stained with EtBr before blotting on to the membrane. (**B**) NotI restriction enzyme map of *S*. *pombe* chromosomes, showing chromosomes I, II, and III (ChI, ChII, and ChIII). (**C**) Spotting assay of a 10-fold serial dilution of cells. We plated *nmt-pot1-aid*, *nmt-pot1-aid rqh1Δ*, *nmt-pot1-aid rqh1-hd*, *nmt-pot1-aid exo1Δ*, *nmt-pot1-aid rqh1Δ exo1Δ* and *nmt-pot1-aid rqh1-hd exo1Δ* on EMM+L or EMM+L plus thiamine and auxin. Before spotting, cells were pre-incubated with 15 μM thiamine for 24 h to reduce *pot1* ^*+*^ expression.

Next, we performed pulsed-field gel electrophoresis (PFGE) to investigate the effects on NotI-digested chromosome-end fragments. Previously, we showed that most of the chromosome-end fragments in the *nmt-pot1-aid* strain, namely I, L, and M [20], disappear following Pot1 shut-off after 12 h incubation and that deletion of *rqh1* ^*+*^ has no effect on this disappearance [15]. Similarly, in the present study, the deletion of *exo1* ^*+*^ had no effect on this disappearance, but concomitant deletion of *rqh1* ^*+*^ and *exo1* ^*+*^ strongly suppressed the loss of signal in I, L, and M chromosome-end fragments (Fig 3A and 3B). These data provide further support for the hypothesis that Rqh1 and Exo1 play redundant roles in the resection of uncapped telomeres. Following Pot1 shut-off, growth of the *nmt-pot1-aid* strain at 12 h was significantly improved with concomitant deletion of *rqh1* ^*+*^ and *exo1* ^*+*^ (Fig 3A). To test the possibility whether cell divisions affected the PFGE results at 12 h in the *nmt-pot1-aid rqh1Δ exo1Δ* strain, we prevented cell division by adding carbendazim (MBC), which arrests the cell cycle at metaphase, at time point 0 h [21]. We found that cell divisions did not affect the signals at the chromosome-end fragments in the *nmt-pot1-aid rqh1Δ exo1Δ* strain at 12 h (Fig 3A 12+MBC). These results suggest that suppression of chromosome-end fragment loss in the *nmt-pot1-aid rqh1Δ exo1Δ* strain at12 h is not attributable to cell divisions. The presence of the L+I band in the *nmt-pot1-aid rqh1Δ exo1Δ* strain at time point 48 h implies that an unknown nuclease (or nucleases) may be involved in resection of uncapped telomere even in the absence of both Rqh1 and Exo1, and which can also fuse telomere ends via single-strand annealing (SSA). This finding is consistent with our previous data, which showed that chromosome ends can be fused by SSA in the *pot1Δ rqh1Δ exo1Δ* triple mutant [15].

Next, we examined the viability of the *nmt-pot1-aid* strain following Pot1 shut-off. The *nmt-pot1-aid* strain lost viability in the presence of both thiamine and auxin (Fig 3C). Single deletion of *rqh1* ^*+*^ suppressed the loss of viability slightly, whereas that of *exo1* ^*+*^ did not cause any suppression. Importantly, concomitant deletion of *rqh1* ^*+*^ and *exo1* ^*+*^ suppressed the loss of viability significantly. These results suggest that the growth suppression observed with concomitant deletion of *rqh1* ^*+*^ and *exo1* ^*+*^ after Pot1 shut-off correlates with the resection of uncapped telomeres.

### Double deletion of *rqh1* ^*+*^ and *exo1* ^*+*^ suppresses RPA foci generation at the uncapped telomere

**Fig 4:**
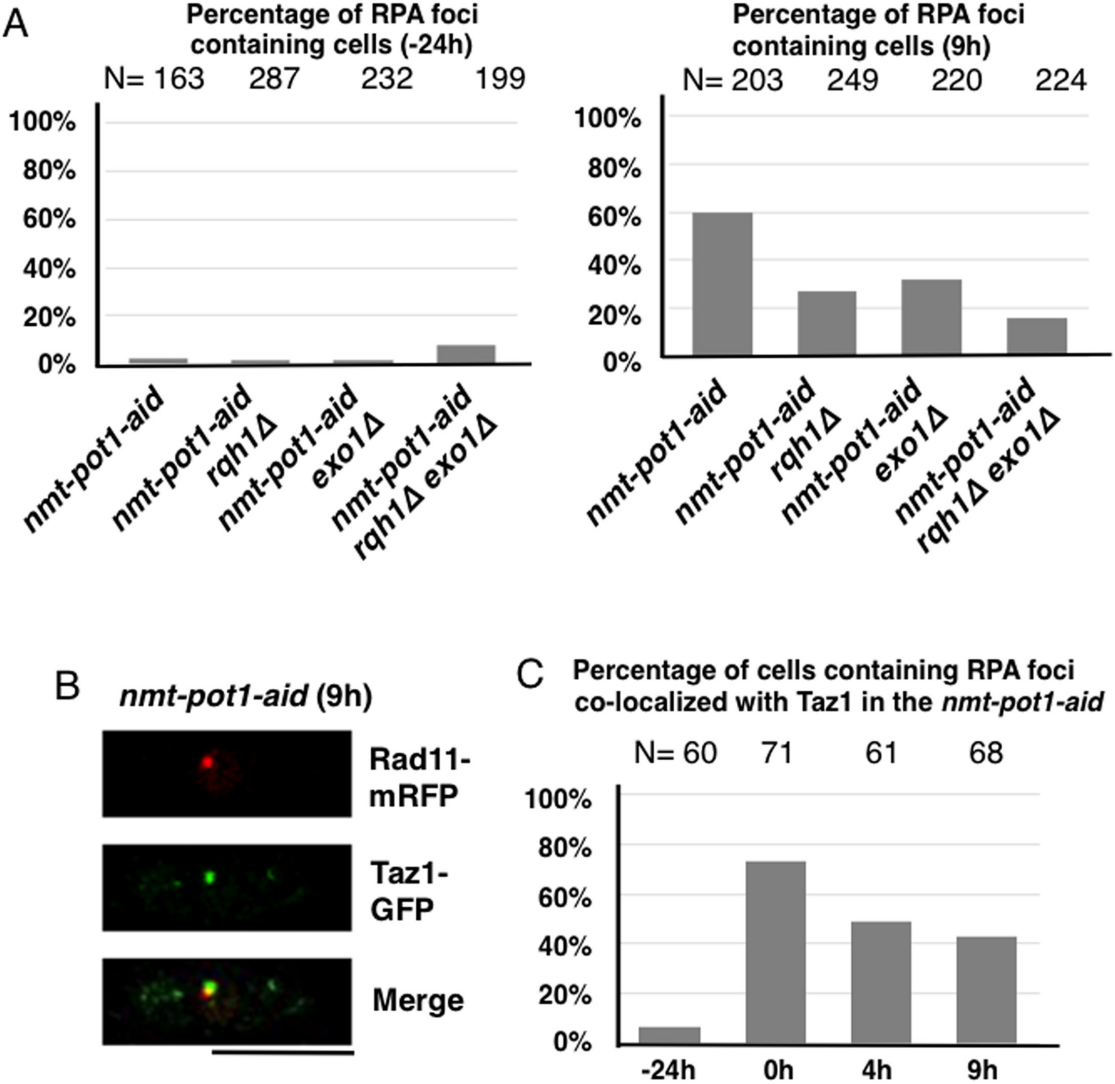
Double deletion of *rqh1* ^*+*^ and *exo1* ^*+*^ suppresses RPA foci generation at the uncapped telomere. (**A**) Percentages of RPA (Rad11) foci containing cells at time points −24 h and 9 h are shown. The *nmt-pot1-aid*, *nmt-pot1-aid rqh1Δ*, *nmt-pot1-aid exo1Δ*, and *nmt-pot1-aid rqh1Δ exo1Δ* strains, with RPA (Rad11) endogenously tagged with mRFP, were analyzed. Cells were incubated as described in Fig 1. The y-axis indicates the percentage of RPA (Rad11) foci-containing cells. The numbers of cells examined (N) are shown at the top. (**B**) Merged microscopic images showing Rad11-mRFP (red) and Taz1-GFP (green) of the *nmt-pot1-aid* strain after 9 h incubation with thiamine and auxin. The large subunit of RPA (Rad11) and Taz1 were endogenously tagged with mRFP and GFP, respectively in the *nmt-pot1-aid* strain. The bar under the diagram represents 10 μm. (**C**) Percentages of RPA (Rad11) foci co-localized with Taz1 foci in the *nmt-pot1-aid* strain used in (B) are shown. Cells were incubated as described in Fig 1. The y-axis indicates the percentages of cells containing RPA (Rad11) foci co-localized with Taz1 foci. The numbers of cells examined (N) are shown at the top.

Replication protein A (RPA) is known to bind to the single-stranded DNA generated by resection at DSBs [22, 23]. We examined the generation of RPA foci in the *nmt-pot1-aid* strain following Pot1 shut-off to investigate the resection of uncapped telomeres. Rad11 (a large subunit of RPA)-mRFP-expressing cells were used to quantify RPA foci-containing cells [15]. We found that most of the cells did not show RPA foci before Pot1 shut-off at time point −24 h (Fig 4A). The percentage of cells containing RPA foci increased in the *nmt-pot1-aid* strain following Pot1 shut-off (9 h) as compared to the −24 h data (Fig 4A). About 50% of these RPA foci co-localized with Taz1-GFP (a telomere marker) [24] at time point 9 h (Fig 4B and 4C), indicating that about 50% (could be more as discussed below) of the RPA foci were produced at telomeres at 9 h. We also found that single deletion of either *rqh1* ^*+*^ or *exo1* ^*+*^ reduced the percentage of cells containing RPA foci at 9 h. Concomitant deletion of *rqh1* ^*+*^ and *exo1* ^*+*^ further reduced this percentage (Fig 4A). These results suggest that the extent of telomere resection is correlated to the percentage of RPA foci-containing cells. We also quantitated the percentage of cells containing RPA foci co-localized with Taz1 in the *nmt-pot1-aid* strain before (−24 h) and after Pot1 shut-off (0, 4, and 9 h)(Fig 4C). Most of the cells had RPA foci that did not co-localize with Taz1 at −24 h, showing that the telomere was capped. More than 70% of the cells had RPA foci co-localized with Taz1 at time point 0 h. This suggests that about 70% of the telomeres were uncapped and telomere resection was initiated at time point 0 h. The percentage of cells containing RPA foci co-localized with Taz1 in the *nmt-pot1-aid* strain decreased at 4 h and 9 h after addition of thiamine and auxin as compared to 0 h (Fig 4C). It is likely that a larger number of uncapped telomeres are resected at 4 and 9 h after addition of thiamine and auxin, as compared to that at 0 h (Figs 1 and 2). This could reduce the quantity of available double-stranded telomere DNA that could be bound by Taz1. Therefore, we assume that some of the RPA foci that did not show Taz1 foci at 4 h and 9 h were still produced at chromosome ends.

### Dna2 and Exo1 resect uncapped telomere redundantly following Pot1 shut-off

**Fig 5:**
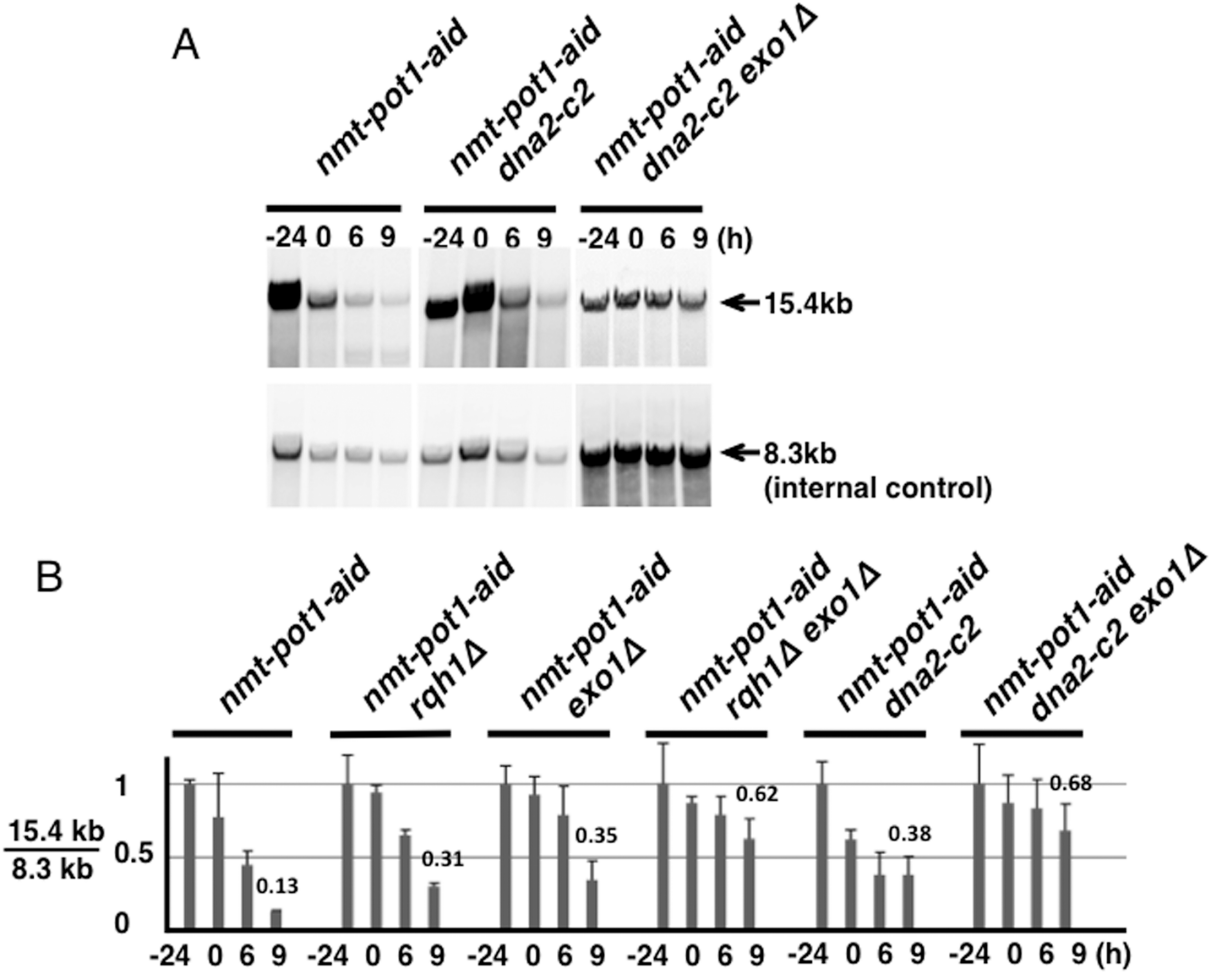
Quantitation of telomere resection following Pot1 shut-off shows that both Dna2 and Exo1 resect uncapped telomere, but redundantly. (**A**) Telomere resection in the *nmt-pot1-aid*, *nmt-pot1-aid dna2-c2* and *nmt-pot1-aid dna2-c2 exo1Δ* strains was analyzed using Southern hybridization as described in Fig 2B. (**B**) The intensity of the 15.4-kb bands in the *nmt-pot1-aid dna2-c2* and *nmt-pot1-aid dna2-c2 exo1Δ* strains was quantitated as described in Fig 2C. For comparison, normalized quantitation values of the telomere loss at time point 9 h are shown above the bars. The same data of the *nmt-pot1-aid*, *nmt-pot1-aid rqh1Δ*, *nmt-pot1-aid exo1Δ*, and *nmt-pot1-aid rqh1Δ exo1Δ* shown in Fig 2C are shown here, for comparison.

It has been reported that in *S*. *cerevisiae*, Dna2 nuclease is responsible for resection at both DSB and uncapped telomeres after DNA unwinding by Sgs1 [10, 17]. The *S*. *pombe dna2-c2* mutant is defective for the production of telomere overhangs in the wild-type and *taz1* disruptant [25]. Therefore, we constructed a *dna2-c2* single mutant and a *dna2-c2 exo1Δ* double mutant with an *nmt-pot1-aid* strain background and examined telomere loss after Pot1 shut-off. At a semi-permissive temperature (30°C), the telomere-loss phenotypes of the *dna2-c2* single mutant and *dna2-c2 exo1Δ* double mutant with an *nmt-pot1-aid* strain background were very similar to those of a *rqh1* single mutant and a *rqh1Δ exo1Δ* double mutant, respectively (Figs 2 and 5). These data indicate that *S*. *pombe* Dna2 is involved in the resection of uncapped telomeres and Dna2 and Exo1 function redundantly.

## Discussion

The resection of uncapped telomeres is well-studied in *S*. *cerevisiae* [26]; however, it is currently unclear whether the mechanisms of the resection in *S*. *cerevisiae* are conserved in other organisms such as *S*. *pombe*. Here, we investigated the contributions of Exo1, Rqh1, and Dna2 to the resection of uncapped telomeres in *S*. *pombe*. Since only surviving *S*. *pombe* cells that lost telomeric DNA were obtained after the loss of the Pot1 plasmid in both the *pot1Δ* single mutant and the *pot1Δ rqh1Δ exo1Δ* triple mutant [12, 15], it was impossible to investigate the specific contributions of Rqh1 and Exo1 in the telomere resection when the *pot1Δ rqh1Δ exo1Δ* triple mutant was used. In contrast, conditional Pot1 shut-off could be used to monitor the time course of telomere loss, which would allow us to investigate the possible contributions of Rqh1 and Exo1 in the telomere resection. Telomeres can be uncapped using the ts *pot1* allele to inactivate Pot1 function conditionally [13], Recently, a *pot1* ts strain was used to identify genes affecting telomere defects, and Exo1 was identified as a suppressor of the *pot1* ts allele [14]. Thus, the ts mutant is very useful to turn off protein function quickly [26]. However, in *S*. *pombe*, the DNA damage checkpoint at high temperature (40°C) is suggested to be different from that at 30°C [27]. To exclude the possibility (however small) that the mechanism of resection of uncapped telomeres at high temperature (36°C) is different from that at 30°C, we attempted to study the resection without a temperature shift.

First, we constructed the *nmt-pot1* strain, in which the promoter of *pot1* ^*+*^ is replaced by the *nmt81* promoter, to shut off *pot1* ^+^ expression by addition of thiamine. This resulted in disappearance of 50% of the telomere signal after 33 h incubation in the presence of thiamine (S1 Fig). Next, we combined the *nmt81* promoter and an *aid*-tag in the *nmt-pot1-aid* strain [15]. When we incubated the *nmt-pot1-aid* strain in the presence of thiamine only for 33 h, 60% of the telomere signal disappeared (S1 Fig). In contrast, when we incubated *nmt-pot1-aid* strain in the presence of both thiamine and auxin for a total of 33 h (24 h in the presence of thiamine and 9 h in the presence of both thiamine and auxin), 70% of the telomere signal disappeared (S1 Fig). These results suggest that the *nmt-pot1-aid* strain is superior to the *nmt-pot1* strain to demonstrate loss of a telomere signal. However, the *aid*-tag itself reduces the stability of Pot1, since the *nmt-pot1-aid* strain lost telomeres to a greater extent than the *nmt-pot1* strain, even in the absence of auxin after Pot1 shut-off by addition of thiamine.

Using the *nmt-pot1-aid* strain background, we found that the loss of a 15.4-kb telomere fragment in the *nmt-pot1-aid* strain after Pot1 shut-off was alleviated by concomitant deletion of *rqh1* ^*+*^ and *exo1* ^*+*^ (Fig 2). One reason for this band disappearing could be due to telomere fusion without resection, which would produce about 30-kb fragment when digested by BglII. However, we never observed this 30-kb fragment in all strains used for Pot1 shut-off (S2 Fig and data not shown), indicating that the reason for the 15.4-kb band disappearing was not due to telomere fusion without resection. Telomere-fusion band could be also produced after telomere resection and SSA, which can produce about 8-kb fragment as detected in *pot1Δ* strain (S2 Fig). However, we did not observe an 8-kb band after 9 h incubation in the presence of thiamine and auxin post Pot1 shut-off with a *dna2-c2* and *dna2-c2 exo1Δ* background (S2 Fig) and with all strains we used for Pot1 shut-off including the *nmt-pot1-aid rqh1Δ*, *nmt-pot1-aid exo1Δ* and *nmt-pot1-aid rqh1Δ exo1Δ* strains, but not the *nmt-pot1-aid* strain (S2 Fig and data not shown). Therefore, the disappearance of the 15.4-kb band was not due to telomere fusion after resection except for the *nmt-pot1-aid* strain. We noted multiple, new, weak bands after Pot1 shut-off in the *nmt-pot1-aid* strain only (S2 Fig). Although we have no direct evidence, we assume that some of these bands could be telomere-fusion bands that were produced after telomere resection, since we noted an increase in the percentage of cells that have RPA foci co-localized with Taz1 after Pot1 shut-off in the *nmt-pot1-aid* strain at time point 0 h compared to −24 h (Fig 4C). This suggests that the telomere end was resected after Pot1 shut-off. SSA could occur at 5 different homology regions (H1 to H5) [28], which would produce different lengths of fusion bands. This is consistent with our data, which showed different lengths of new bands after Pot1 shut-off in the *nmt-pot1-aid* strain (S2 Fig), further suggesting that some of these bands could be produced after the resection. However, further study is required to understand the nature of these new bands.

Our data suggest that both Rqh1 and Exo1 contribute to the resection of uncapped telomeres, but do so redundantly. Importantly, uncapped telomeres were significantly resected even in the absence of Exo1, which suggests that an Rqh1-mediated pathway can resect uncapped telomeres in the absence of Exo1. Although this finding shows that the redundant pathways with Exo1 and Sgs1/Rqh1 in the resection of uncapped telomere are conserved between *S*. *cerevisiae* and *S*. *pombe*, it is different in the way that Exo1 and Sgs1 pathways play a major role in the resection of uncapped telomeres in *S*. *cerevisiae*, but other unknown pathway(s) contribute to the resection in *S*. *pombe* [10].

We found that the *nmt-pot1-aid* strain lost its viability in the presence of thiamine and auxin (Fig 3C). This loss of viability was suppressed by the concomitant deletion of both *rqh1* ^*+*^ and *exo1* ^*+*^, which suggests that the viability correlates with the extent of resection of uncapped telomeres. Although the *exo1Δ* cells exhibited a greater long-range resection defect than the *rqh1Δ* cells after Pot1 shut-off (Fig 2C), the growth suppression resulting from the *rqh1* ^*+*^ deletion was greater than that due to the *exo1* ^*+*^ deletion (Fig 3C). Growth could be affected by checkpoint activation at the uncapped telomere. Rqh1 could unwind the secondary structure of the telomere overhang [29], which may affect the RPA binding required for DNA-damage checkpoint activation [30]. Therefore, Rqh1 may contribute to activation of the DNA-damage checkpoint to a greater extent than Exo1, which could result in growth suppression by the *rqh1* ^*+*^ deletion. Consistently, *exo1Δ* cells showed more RPA foci than *rqh1Δ* cells after Pot1-shut-off (Fig 4A 9 h).

Interestingly, mutation in the helicase domain in *rqh1* (*rqh1-hd)* resulted in greater suppression in the loss of viability, compared to that observed for null mutation of the *rqh1* ^*+*^ gene (*rqh1Δ*). This implies that the binding of helicase-dead Rqh1 to telomere ends may inhibit resection by nucleases such as Exo1. Indeed, the extent of long-range resection in the 4.0-kb fragment, as observed in the *rqh1-hd* cells, was slightly less than that observed in the *rqh1Δ* cells, which shows similar phenotype with the *exo1Δ* cells, following Pot1 shut-off (Fig 2C). This is consistent with finding in *S*. *cerevisiae*, where helicase-dead Sgs1 inhibits Exo1-dependent resection [31]. Another plausible explanation for greater suppression of the loss of viability is that telomere-bound, helicase-dead Rqh1 may physically block recruitment of DNA-damage checkpoint proteins to the uncapped telomere, which could in turn inhibit activation of the DNA-damage checkpoint.

The percentage of RPA foci-containing cells of the *nmt-pot1-aid* strain increased following Pot1 shut-off (Fig 4). The proportion of RPA foci-containing cells among cells with the concomitant deletion of both *rqh1* ^*+*^ and *exo1* ^*+*^ was found to decrease, suggesting that production of RPA foci correlates with the extent of resection at uncapped telomeres. Most of the RPA foci seen at −24 h in the *nmt-pot1-aid* strain did not co-localize with Taz1 (Fig 4C). We noted a greater quantity of RPA foci at −24 h with an *rqh1Δ exo1Δ* background as compared to the wild-type background at −24 h (Fig 4A). This increase in RPA foci with an *rqh1Δ exo1Δ* background at −24 h could be due to increase in the spontaneous DNA damage due to the defect in DNA resection from an *rqh1Δ exo1Δ* background [6]. Therefore, there may be more spontaneous, non-telomeric, RPA foci in the *rqh1Δ exo1Δ* background even after Pot1 shut-off, which could cause over-estimation of the percentage of RPA foci produced at the telomere in the *rqh1Δ exo1Δ* background at 9 h.

Mouse Apollo, which shows 5′ to 3′ exonuclease activity in vitro, is thought to be involved in the processing of the leading-end telomere formed during DNA replication [32–34]. Mouse Exo1 has also been linked to the telomerase-independent generation of telomere overhangs following DNA replication [35]. In a previous study, deletion of both Apollo and Exo1 had an additive effect on telomerase-independent generation of the leading-end telomere overhang, suggesting that they play independent roles in generating telomere overhangs following DNA replication [36]. Telomere uncapping by deletion of mouse *Pot1b* also causes Exo1- and Apollo-dependent resection [36]. Taken together, our results show that the role of Exo1 in the resection of uncapped telomeres is conserved in *S*. *cerevisiae*, *S*. *pombe*, and mammals. Although human BLM (RecQ helicase) is thought to be involved in the processing of DSBs [16, 37], the role of mammalian BLM in the processing of uncapped telomeres remains unclear.

In *S*. *cerevisiae*, Sgs1-Dna2 and Exo1 resect DSB redundantly [17]. *S*. *pombe* Dna2 is also reported to be involved in the resection at DSB, at least in the *exo1Δ* background [7]. Moreover, we previously showed that the *S*. *pombe dna2-c2* mutant is defective in the generation of telomere overhangs in the wild-type and *taz1* disruptant, at a semi-permissive temperature [25]. We consistently found that Exo1 and Dna2 resected uncapped telomeres redundantly after Pot1 shut-off at a semi-permissive temperature (30°C) (Fig 5). The extent of telomere resection in the *rqh1Δ exo1Δ* double mutant was very similar to that in the *dna2-c2 exo1Δ* double mutant. Moreover, human DNA2 and BLM (an Rqh1 homologue) function together to repair DSBs [37], suggesting that *S*. *pombe* Dna2 functions together with Rqh1 in telomere-end resection.

In conclusion, this study is the first to show that Exo1 and Rqh1-Dna2 contribute to the resection of uncapped telomeres redundantly in *S*. *pombe*. We also demonstrated that *nmt-pot1-aid* is an important model strain to investigate the role of helicases and nucleases in the resection of uncapped telomeres and, potentially, in DSB-end resection.

## Materials and Methods

The strains used in this study are listed in Table 1. The *nmt-pot1-aid exo1Δ*, *nmt-pot1-aid rqh1-hd exo1Δ*, *nmt-pot1-aid rqh1Δ exo1Δ*, *nmt-pot1-aid rqh1-hd exo1Δ*, *nmt-pot1-aid dna2-c2Δ* and *nmt-pot1-aid dna2-c2 exo1Δ* strain were created by mating strains carrying *exo1Δ*, *rqh1Δ*, *rqh1-hd*, or *dna2-c2* with those carrying *nmt-pot1-aid* containing strains [15, 25, 38]. To tag the Rad11 protein in *nmt-pot1-aid* cells with monomeric red fluorescent protein (mRFP) at the C-terminus, pFA6a-mRFP-natMX6-rad11 was linearized with NspV and used for transformation [39]. The *nmt-pot1-aid* strain containing *rad11-mRFP*, *Taz1-GFP*, and TN177, was created by mating strain NH013 with strain jcf4523. Cells were grown in YEA medium (0.5% yeast extract, 3% glucose, and 40 μg/ml adenine) or Edinburgh minimal medium (EMM), with required supplements, at 30°C [40]. For spot assays, cells were grown to a concentration of 1 × 10^7^ cells/ml in YEA. Serial dilutions (1:10) were prepared, and 4-μl aliquots were spotted onto plates.

**Table 1:** *S*. *pombe* Strains Used in This Study.

Strain	Genotype	Source
NH001	*h* ^*-*^ *leu1-32 ura4-D18 pot1*::*sup3-5-nmt81-pot1* ^*+*^ *-IAA17*::*ura4* ^*+*^ *ade6*::*ade6* ^*+*^ *-Padh15-skp1-AtTIR1-2NLS-9myc*	[15]
NH002	*h* ^*-*^ *ura4-D18 pot1*::*sup3-5-nmt81-pot1* ^*+*^ *-IAA17*::*ura4* ^*+*^ *ade6*::*ade6* ^*+*^ *-Padh15-skp1-AtTIR1-2NLS-9myc rqh1*::*hphMX6*	[15]
KTA050	*h* ^*+*^ *leu1-32 ura4-D18 pot1*::*sup3-5-nmt81-pot1* ^*+*^ *-IAA17*::*ura4* ^*+*^ *ade6*::*ade6* ^*+*^ *-Padh15-skp1-AtTIR1-2NLS-9myc exo1*::*aur1*	This study
KTA49	*h* ^*-*^ *ura4-D18 pot1*::*sup3-5-nmt81-pot1* ^*+*^ *-IAA17*::*ura4* ^*+*^ *ade6*::*ade6* ^*+*^ *-Padh15-skp1-AtTIR1-2NLS-9myc exo1*::*aur1 rqh1*::*hphMX6*	This study
KTA013	*h* ^*+*^ *leu1-32 ura4-D18 pot1*::*sup3-5-nmt81-pot1* ^*+*^ *-IAA17*::*ura4* ^*+*^ *ade6*::*ade6* ^*+*^ *-Padh15-skp1-AtTIR1-2NLS-9myc rqh1-K547A*	This study
KTA148	*h* ^*-*^ *ura4-D18 pot1*::*sup3-5-nmt81-pot1* ^*+*^ *-IAA17*::*ura4* ^*+*^ *ade6*::*ade6* ^*+*^ *-Padh15-skp1-AtTIR1-2NLS-9myc exo1*::*aur1 rqh1-K547A*	This study
NH007	*h* ^*+*^ *ura4-D18 pot1*::*sup3-5-nmt81-pot1* ^*+*^ *-IAA17*::*ura4* ^*+*^ *ade6*::*ade6* ^*+*^ *-Padh15-skp1-AtTIR1-2NLS-9myc exo1*::*aur1 dna2-c2*	This study
LN006	*h* ^*-*^ *ura4-D18 pot1*::*sup3-5-nmt81-pot1* ^*+*^ *-IAA17*::*ura4* ^*+*^ *ade6*::*ade6* ^*+*^ *-Padh15-skp1-AtTIR1-2NLS-9myc dna2-c2*	This study
NH012	*h* ^*-*^ *leu1-32 ura4-D18 pot1*::*sup3-5-nmt81-pot1* ^*+*^ *-IAA17*::*ura4* ^*+*^ *ade6*::*ade6* ^*+*^ *-Padh15-skp1-AtTIR1-2NLS-9myc rad11-mRFP*::*natMX6*	This study
NH013	*h* ^*-*^ *ura4-D18 pot1*::*sup3-5-nmt81-pot1* ^*+*^ *-IAA17*::*ura4* ^*+*^ *ade6*::*ade6* ^*+*^ *-Padh15-skp1-AtTIR1-2NLS-9myc rqh1*::*hphMX6 rad11-mRFP*::*natMX6*	This study
TANA116	*h* ^*+*^ *leu1-32 ura4-D18 pot1*::*sup3-5-nmt81-pot1* ^*+*^ *-IAA17*::*ura4* ^*+*^ *ade6*::*ade6* ^*+*^ *-Padh15-skp1-AtTIR1-2NLS-9myc exo1*::*aur1 rad11-mRFP*::*natMX6*	This study
TN175	*h* ^*-*^ *ura4-D18 pot1*::*sup3-5-nmt81-pot1* ^*+*^ *-IAA17*::*ura4* ^*+*^ *ade6*::*ade6* ^*+*^ *-Padh15-skp1-AtTIR1-2NLS-9myc exo1*::*aur1 rqh1*::*hphMX6 rad11-mRFP*::*natMX6*	This study
TN177	*h* ^*-*^ *ura4-D18 pot1*::*sup3-5-nmt81-pot1* ^*+*^ *-IAA17*::*ura4* ^*+*^ *ade6*::*ade6* ^*+*^ *-Padh15-skp1-AtTIR1-2NLS-9myc rad11-mRFP*::*natMX6 Taz1-GFP*::*kanMX6*	This study
jcf4523	*h* ^*-*^ *ade6-M210 leu1-32 ura4-D18 taz1-GFP*::*kanMX*	J. Cooper
JY741	*h* ^*-*^ *leu1-32 ura4-D18 ade6-M216*	M.Yamamoto
KTA045	*h* ^*+*^ *leu1-32 ura4-D18 ade6-M210 pot1*::*kanMX6 rqh1-K547A rad11-mRFP*::*natMX6 pREP41-Top3-Y330F*	[15]

### Measurement of telomere length

Telomere length was measured using Southern hybridization, as previously described [41], using AlkPhos Direct Labeling Module (GE Healthcare). For the probes shown in Fig 1, we used a telomere-associated sequence plus telomere fragment, digested with EcoRI derived from pNSU70 [42] and NBS1. For quantitation of telomere resection (Fig 2), 3 probes designated as I, II, and III were used to detect 15.4-, 4.0-, and 8.3-kb BglII-digested fragments, respectively. Primers used for amplification of the probes I, II, III, and NBS1 are listed in Table 2.

**Table 2:** Primer list.

Name	Sequence
probe NBS1 top	5’-cattatggccgtattgtacgta-3’
probe NBS1 bot	5’-catacttctccagtatgcact-3’
probe I top	5’-cactgttcttaagtattgttcg-3’
probe I bot	5’-gcataaagatggtacttcaa-3’
probe II top	5’-gcatcggaagctgctttcagc-3’
probe II bot	5’-gttacgaagtctcccttaac-3’
probe III top	5’-gcatacccgggttaaaagtgaaacttgagatcat-3’
probe III bot	5’-gcatagtcgacaatgtggataattgaggctg-3’

### Pulsed-field gel electrophoresis (PFGE)

We performed PFGE as described by Baumann et al. [43]. For the detection of NotI-digested chromosomes, we fractionated NotI-digested *S*. *pombe* chromosomal DNA on a 1% agarose gel with a 0.5× TBE buffer (50 mM Tris-HCl, 5 mM boric acid, and 1 mM ethylenediaminetetraacetic acid, pH 8.0) at 14°C. We used the CHEF Mapper PFGE system, operated for 24 h at 6 V/cm (200 V) with a pulse time of 60 to 120 s. DNA was visualized by staining with 1 μg/ml EtBr for 30 min.

### Microscopy

Microscopic images of living cells were obtained as described by Nanbu et al. [15], using an AxioCam digital camera (Zeiss) connected to an Axio Observer.Z1 microscope (Zeiss) with a Plan-Apochromat 63× objective lens (numerical aperture, 1.4).

## Supporting Information

S1 Fig Replacing the *pot1* ^*+*^ promoter with the *nmt81* promoter is inadequate to reduce the telomere signal. **(A)** Experimental design. Experiments were performed as shown in Fig 1A, except that thiamine was added as indicated. **(B)** Telomere length in the wild-type (JY741), *nmt-pot1* (RM002 *h* ^*-*^ *nmt81-pot1* ^*+*^::*sup3-5 leu1-32 ura4-D18 ade6-704*), and *nmt-pot1-aid* (NH001) strains was analyzed using Southern hybridization as described in Fig 1B. Cells were cultured as described in Fig 1B, except that the wild-type, *nmt-pot1*, and *nmt-pot1-aid* strains were incubated with thiamine as indicated. The normalized quantification value of the telomere band is shown below the Southern hybridization data, as in Fig 1B.
(TIF)

S2 Fig Telomere-fusion band was not detected in the *nmt-pot1-aid dna2-c2* and *nmt-pot1-aid dna2-c2 exo1Δ* strains following Pot1 shut-off. The full range of Southern hybridization data used in Fig 5A with the data of *pot1Δ* are shown. The *pot1Δ* strain was incubated without thiamine and auxin. The probe I used in Fig 2 was used. The BglII sites of the terminal region on chromosomes I and II are shown [28]. The telomere-fusion band produced by SSA after the telomere resection in the *pot1Δ* strain is shown by an arrowhead, and the 15.4-kb telomere band and 2 non-specific bands are shown by arrows. New bands detected in the *nmt-pot1-aid* strain after Pot1 shut-off are shown by dashed arrows. New bands were also not detected in the *nmt-pot1-aid rqh1Δ*, *nmt-pot1-aid exo1Δ* and *nmt-pot1-aid rqh1Δ exo1Δ* strains (data not shown).
(TIF)
