# Supplementary figures and images for: Fission Yeast Exo1 and Rqh1-Dna2 Redundantly Contribute to Resection of Uncapped Telomeres
Source: PLoS One. 2015 Oct 14;10(10):e0140456. doi: 10.1371/journal.pone.0140456 (PMC4605587; doi:10.1371/journal.pone.0140456)

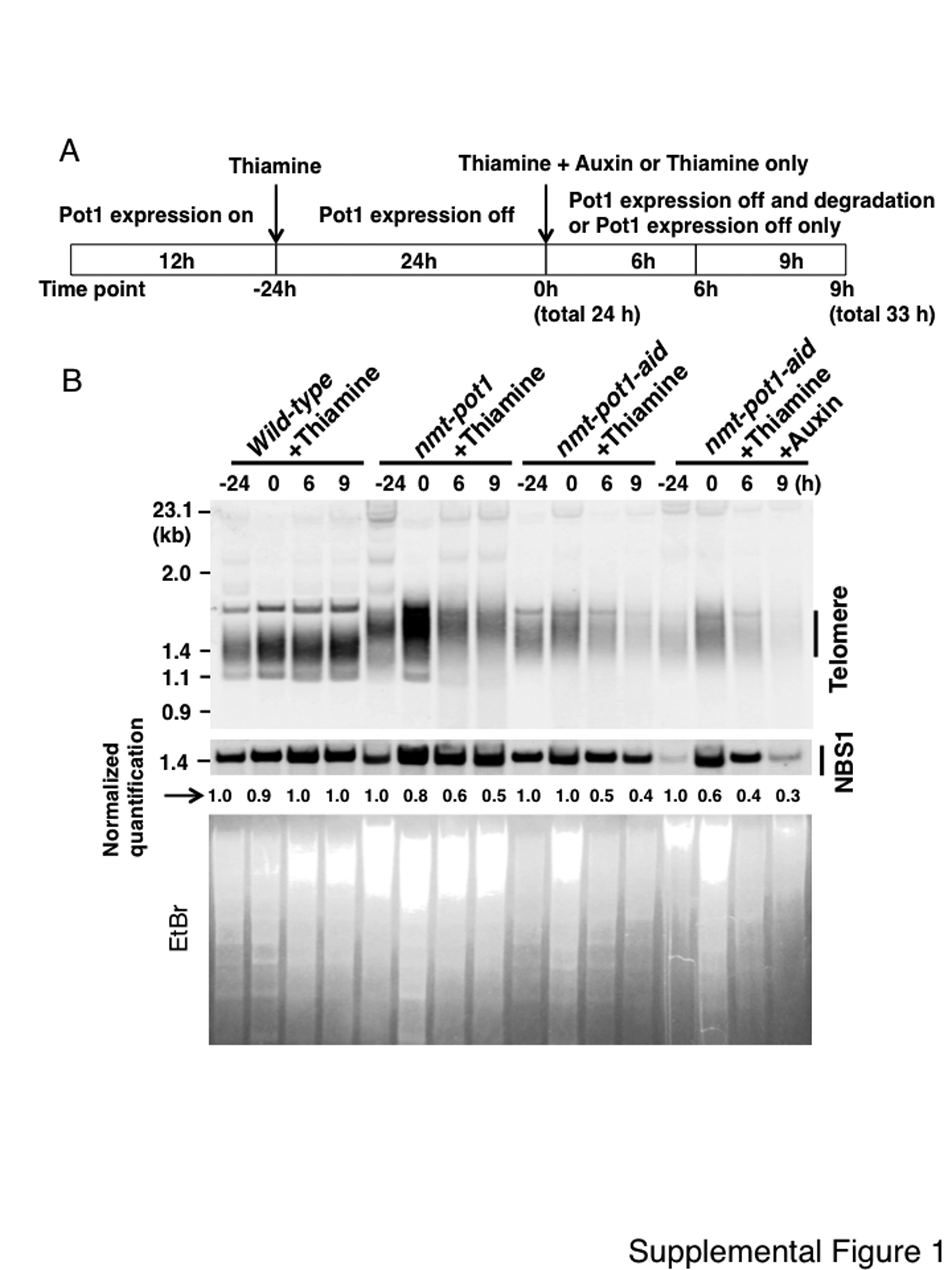

Supplement: S1 Fig — (A) Experimental design. Experiments were performed as shown in Fig 1A, except that thiamine was added as indicated. (B) Telomere length in the wild-type (JY741), nmt-pot1 (RM002 h - nmt81-pot1 +::sup3-5 leu1-32 ura4-D18 ade6-704), and nmt-pot1-aid (NH001) strains was analyzed using Southern hybridization as described in Fig 1B. Cells were cultured as described in Fig 1B, except that the wild-type, nmt-pot1, and nmt-pot1-aid strains were incubated with thiamine as indicated. The normalized quantification value of the telomere band is shown below the Southern hybridization data, as in Fig 1B. (TIF) [file pone.0140456.s001.tif]

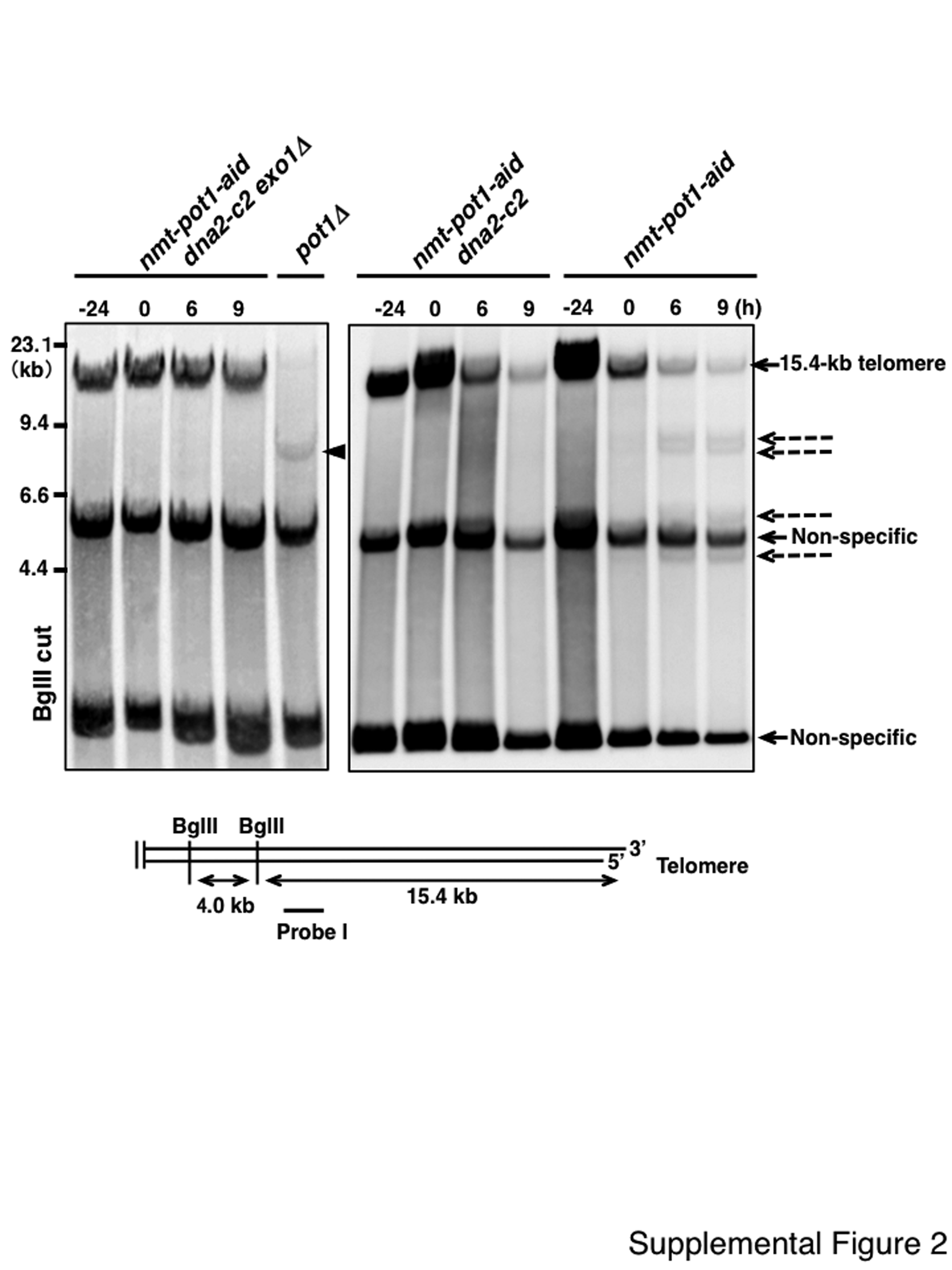

Supplement: S2 Fig — The full range of Southern hybridization data used in Fig 5A with the data of pot1Δ are shown. The pot1Δ strain was incubated without thiamine and auxin. The probe I used in Fig 2 was used. The BglII sites of the terminal region on chromosomes I and II are shown [28]. The telomere-fusion band produced by SSA after the telomere resection in the pot1Δ strain is shown by an arrowhead, and the 15.4-kb telomere band and 2 non-specific bands are shown by arrows. New bands detected in the nmt-pot1-aid strain after Pot1 shut-off are shown by dashed arrows. New bands were also not detected in the nmt-pot1-aid rqh1Δ, nmt-pot1-aid exo1Δ and nmt-pot1-aid rqh1Δ exo1Δ strains (data not shown). (TIF) [file pone.0140456.s002.tif]
